# A Portable, Label-Free, Reproducible Quartz Crystal Microbalance Immunochip for the Detection of Zearalenone in Food Samples

**DOI:** 10.3390/bios11020053

**Published:** 2021-02-19

**Authors:** Shengmiao Liu, Xinyu Liu, Qianwen Pan, Zhihan Dai, Mingfei Pan, Shuo Wang

**Affiliations:** 1 State Key Laboratory of Food Nutrition and Safety, Tianjin University of Science & Technology, Tianjin 300457, China; lsm@mail.tust.edu.cn (S.L.); liuxy@mail.tust.edu.cn (X.L.); pqw@mail.tust.edu.cn (Q.P.); dzh@mail.tust.edu.cn (Z.D.); s.wang@tust.edu.cn (S.W.); 2 Key Laboratory of Food Nutrition and Safety, Ministry of Education of China, Tianjin University of Science & Technology, Tianjin 300457, China

**Keywords:** zearalenone, quartz crystal microbalance, portable and reproducible immunochip, label free detection

## Abstract

This research reports a portable immunochip, based on quartz crystal microbalance (QCM) for label-free, low-cost qualitative detection of zearalenone (ZEN) in food samples. The experimental parameters in the functionalization and working process were evaluated in detail, in order to achieve a high accuracy and sensitivity. Under optimal conditions, the ZEN concentration at an inhibition ratio of 50% and 15% of the proposed QCM immunochip achieved 3.41 µg L^−1^ and 0.37 µg L^−1^, respectively. This portable QCM immunochip also exhibited high specificity, no obvious cross-reaction to five structural analogs of ZEN, and showed other mycotoxins. It could finish the whole qualitative measurement within 30 min, showed good stability during the processes of preparation (SD < 5%, *n* = 9), storage (frequency response >90%, in PBS at 4 °C for 15 days), and application (frequency response >90% after being reused 6 times). The developed QCM immunochip obtained accurate and repeatable recovery results in ZEN analysis in the chosen food samples (corn, wheat flour, soy sauce, and milk), which had a high correlation (R^2^ = 0.9844) with that achieved by the HPLC–MS/MS method. In short, this work developed a portable, stable, and reproducible QCM immunochip that could be used for rapid, low-cost, and sensitively measurement of ZEN content in real food samples.

## 1. Introduction

Zearalenone (ZEN), also known as F-2 toxin, is a secondary metabolite produced by Fusarium, which is a typical food contaminant [1,2]. Due to its structural similarity to endogenous estrogen, ZEN can competitively bind to estrogen receptors, leading to hormonal imbalances in the body that ultimately damage the animal’s reproductive system [3]. There are many types of food that can be easily contaminated by ZEN, including grains like corn, soybeans, sorghum, and their products like oils, sauces, wine, etc. [4,5,6]. ZEN, which can be ingested directly or indirectly through the food chain, not only poisons the human body directly, but can also transmit to the next generation through the placenta or breast milk, posing a serious threat to people’s health [7,8]. In modern medical research, ZEN was proved to have significant immunotoxicity, genetic toxicity, and reproductive toxicity [9,10,11]. Under high exposure and accumulation concentration, ZEN can inhibit the activity of enzymes in the body and stimulate the growth of breast cancer cells. In order to control and reduce the adverse effects of highly toxic ZEN on people’s health, many countries and organizations set limited requirements on the amount of ZEN contaminant in food. The United States: 20–100 µg kg^−1^ in cereal products; European Union: 350 µg kg^−1^ in unprocessed corn products; and China: 60 µg kg^−1^ in cereals and cereal products [12,13]. Therefore, controlling the content of ZEN contaminant in foods could improve the safety of food, which is necessary and significant for protecting people’s health and promoting the trade of related food [14].

At present, the strategies for ZEN pollutant quantitative analysis in food are mainly instrumental analysis, based on chromatographic separation and mass spectrometry [15,16,17], and immunoassays based on antigen–antibody (Ab) specific reaction [18,19]. Chromatographic separation technology could effectively remove the interference of complex food matrix, which gives instrumental analysis significant advantages in detection accuracy and sensitivity. Meanwhile, the highly integrated and automated analytical process make the analysis results highly stable and repeatable [20,21]. However, these methods usually require relatively expensive equipment and skilled operators and they have obvious shortcomings in terms of low cost and rapid screening of a large number of samples. Immunoassays based on specific binding reaction of antigen and Ab, such as ELISA and lateral flow immunochromatographic strips, have low detection cost and are easy to realize on-line and through on-site detection [22,23,24], but are prone to “false positive” results, due to environmental factors or improper operation.

As an important branch of immunoassay, the immunosensing chip does not only have high specificity and sensitivity, but also have the advantages of miniaturization, high portability, and high stability, which is very suitable for rapid and on-site screening of a large number of samples. This provided new strategies for the detection of trace hazardous substances, such as ZEN, in foods. Many efforts are currently directed into exploring new approaches that can improve the sensitivity and simplicity of immunosensors. Among them, constructing a high-performance immunosensing chip by combining them with surface plasmon resonance [25,26], piezoelectric [27], fluorescence [28,29], and other sensitive signals [30,31] was a successful attempt, which attracted widespread attention of researchers in recent years. These immunochips overcome the shortcoming of traditional immunoassay devices that require enzymes or other markers and are prone to false positive results, showing broad application prospects.

**Figure 1:**
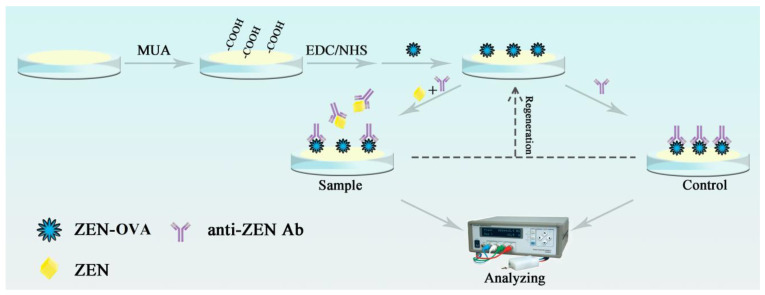
The functionalization and detection process of the developed QCM immunochip.

The oscillating quartz crystal device is such a kind of sensor that uses quartz crystal microbalance (QCM) to monitor the change of resonant frequency caused by the change of surface mass of quartz crystal chip [32,33]. Although QCM analysis is characterized by high precision, good stability, good reproducibility, wide linear range, and low detection limit, constructing QCM immunosensing chip for direct analysis of small molecular analytes at trace amount proved to be difficult [34,35]. Therefore, in this study, a portable and reusable QCM immunosensing chip, based on the immunosuppression format, was developed and applied to the quantitative analysis of trace ZEN contaminant, in actual food samples. The construction process of the QCM immunochip was as follows. First, the conjugate of ZEN–ovalbumin (ZEN–OVA) was covalently conjugated on the surface of QCM chips modified with 11mercaptodecylic acid (MUA), through an amide reaction. The inhibition reaction was carried out by incubating ZEN and ZEN–OVA with anti-ZEN Ab, at a steady concentration. The frequency response generated by the QCM sensing chip was related to the weight change caused by the specific binding of anti-ZEN Ab to ZEN–OVA on the chip surface. Therefore, label-free detection for ZEN contaminant in samples was achieved. Figure 1 showed the functionalization process of the QCM immunochip and its detection process to the ZEN contaminant.

## 2. Materials and Methods

### 2.1. Materials

The analyte ZEN and other mycotoxins including α-zearalenol, β-zearalenol, α-zearalanol, β-zearalanol, zearalanone, aflatoxin B_1_ (AFB_1_), ochratoxin A (OTA), T-2 toxin, fumonisin B_1_ (FB_1_), and deoxynivalenol (DON) were purchased from Sigma-Aldrich (St. Louis, MO, USA), which was individually dissolved into methanol (1.0 mg mL^−1^) and stored at 4 °C. The anti-ZEN monoclonal antibody (anti-ZEN Ab, 1 mg mL^−1^) and the ZEN–ovalbumin conjugate (ZEN–OVA, 2.94 mg mL^−1^) were sourced from Shandong Lvdu Biotechnology Co. Ltd. (Shandong, China). The ethyl-3-(3-(dimethylamino)propyl) carbodiimide (EDC) and *N*-hydroxysuccinimide (NHS) were also sourced from Sigma-Aldrich (St. Louis, MO, USA). The bovine serum albumin (BSA) and MUA were purchased from J&K Scientific Ltd. (Beijing, China), and the other reagents used in the experiment were purchased from the Tianjin Chemical Reagent Factory (Tianjin, China). Food samples including corn powder, wheat flour, soy sauce, and milk were obtained from a local supermarket (Tianjin, China).

A Type-922 QCM from the Princeton Applied Research (Oak Ridge, TN, USA) was used for monitoring the oscillation frequency response. The quartz crystal with Au electrodes on both sides (9 MHz, AT-cut, 0.196 cm^2^) and a flow cell (CL6) to ensure the solution flowed over the crystal chip surface were both purchased from Seiko EG&G (Tokyo, Japan). A precision pump from the Changzhou VCL Fluid Technology (Changzhou, China) was used to load the tested sample solution. A centrifuge machine (Eppendorf, China), extraction cartridges filled with 60-mg filter (*N*-propyl ethylenediamine:C_18_:MgSO_4_ = 3:4:4, m/m/m) (Agela Technologies, Tianjin, China) was applied for sample pretreatment. A high-performance liquid chromatography tandem mass spectrometry system (HPLC–MS/MS, Agilent, California, USA) was used to verify the results of ZEN analysis in selected food products. The separation of sample extract was performed on a ZORBAX SB-C_18_ column (2.1 × 100 mm, 3.5 μm, Agilent, USA) using the mixture of CH_3_COONH_4_ (0.005 mol L^−1^) and acetonitrile as mobile phase, at a flow rate of 0.4 mL min^−1^ at 40 °C.

### 2.2. Pretreatment of the QCM Chip

Before use, the quartz crystal chip surface was washed using freshly prepared “Piranha” solution (30% H_2_O_2_:98% H_2_SO_4_ = 1:3, *v*/*v*) for 3 min, and then rinsed by deionized water and ethanol, and dried under N_2_. The cleaned quartz crystal chip was further mounted in the flow cell and the initial resonance frequency (*f*_0_, Hz) was recorded. First, 100 μL of MUA in ethanol (10 mmol L^−1^) was applied to treat the quartz crystal chip surface to form an oriented terminal carboxy-terminated thiol monolayer through the Au-S bond. A total of 100 μL of the mixture of EDC (0.4 mol L^−1^) and NHS (0.1 mol L^−1^) (1:1, *v*/*v*) was used to replace the –COOH group, using ester groups on the chip surface. Furthermore, the ZEN–OVA conjugate in acetate buffer (29.4 μg mL^−1^, pH 4.5) were uploaded onto the activated chip surface and then incubated at 37 °C for 2 h. Subsequently, the PBS containing 0.1% Tween 20 (50 μL) was used to rinse the chip surface and then dried using N_2_. As a result, the conjugate of ZEN–OVA was immobilized on the QCM chip surface. 

After using BSA (1%, 100 μL) to block the untreated sites, the QCM chip surface was in turn washed by PBS containing 0.1% Tween 20, deionized water, and dried using N_2_ gas. The constructed QCM immunochip was stored under buffered humidity (4 °C), prior to use.

### 2.3. Measuring Procedure for ZEN Contaminant

Different concentrations (0.05, 0.25, 0.5, 1.25, 5.0, 25.0, 50.0, 100.0, and 200.0 µg L^−1^) of ZEN working standard solution were obtained by gradient diluting ZEN stock solution (1.0 mg mL^−1^), using the PBS (pH 7.4) solution. This series of ZEN working solutions were individually incubated with anti-ZEN Ab (100 µg mL^−1^) for 10 min and the mixture was uploaded onto the quartz crystal surface to allow the unbound Ab to react with the immobilized ZEN–OVA conjugate, causing the frequency change (Δ*f = f*_control_ *− f*_test_, *Hz*) of the QCM chip. According to the resulting Δ*f* values, the inhibition ratio at different concentration of ZEN was calculated using the following formula.

$$Inhibition\;ratio\;(\%)=\frac{f\mathrm{control}-f\mathrm{test}}{f\mathrm{control}}\times 100\%$$

where the *f*_control_ stands for the QCM frequency response without the ZEN analyte, and *f*_test_ means the frequency response with different ZEN concentration.

### 2.4. Regeneration of the QCM Immunochip

After each measurement, the QCM immunochip was returned to the original state (Initial frequency) using the regeneration solution, i.e., the regeneration solution was required only to remove the Ab bound to the ZEN–OVA conjugate, without affecting the ZEN–OVA layer on the chip surface. Therefore, the next analysis using such a chip could not be affected. A total of 100 µL of regeneration solutions with different property including 0.1 mol L^−1^ HCl, 0.01 mol L^−1^ Gly-HCl (pH 1.5), SDS (0.5%), and 0.05 mol L^−1^ NaOH were applied onto the chip surface for 5 min for chip regeneration, respectively. After rinsing using PBS (pH 7.4), the QCM immunochip was tested at the base and at a testing frequency of 50.0 μg L^−1^ ZEN concentration, for comparison.

### 2.5. Cross-Reactivity of the QCM Immunochip

The specificity of the developed QCM immunochip for ZEN analysis was evaluated by detecting five ZEN structural analogues (α-zearalenol, β-zearalenol, α-zearalanol, β-zearalanol, and zearalanone), AFB_1_, OTA, FB_1_, and DON at a series of concentrations. According to the value of 50% inhibitory concentration (*IC*_50_) on different analyte, percent cross-reactivity for ZEN analysis using the developed QCM immunochip was calculated using the following formula.

$$Percent\;cross-reactivity\;(CR,\%)\;=\frac{{IC}_{50}\;\mathrm{for}\;\mathrm{ZEN}}{{IC}_{50}\;\;\mathrm{for}\;\mathrm{the}\;\mathrm{other}\;\mathrm{analyte}}$$

### 2.6. Sample Pretreatment and Spiked Method

Corn flour, wheat flour, soy sauce, and milk were chosen as practical food samples for analysis. Each chosen food sample was determined to be free of ZEN, before use. The chosen samples were spiked with ZEN dissolved in methanol (1.0 mg L^−1^) to give three concentration levels of 5.0, 50.0, and 100.0 μg kg^−1^ (μg L^−1^). After thorough mixing and being allowed to stand for 4 h, the mixture was applied to perform a pretreatment process for further research. Two grams (or mL) of each food samples were accurately weighted into a centrifuge cube and mixed with 10.0 mL of methanol/water mixture (3/2, *v*/*v*) to remove the biological molecules. After shaking for 3 min and centrifuging at 3500 rpm at 4 °C, the supernatant obtained was separated, dried, and re-diluted to a suitable volume, for QCM analysis.

## 3. Results and Discussions

### 3.1. Functionalization of the QCM Immunochip

Since the constructed QCM immunochip works in an inhibitory format, it is very important to effectively immobilize the ZEN–OVA conjugates on the surface of QCM immunochip, which affects the stability and reusability of the QCM immunochip. The used MUA can introduce carboxyl groups on the Au surface of chip, through AU-S self-assembly. These carboxyl groups can form a covalent bond with the amino group of OVA, thereby immobilizing ZEN–OVA on the chip surface. At the same time, enough ZEN–OVA conjugates stably immobilized on the chip surface makes a crucial contribution to improving the detection accuracy, sensitivity, and stability of the constructed QCM immunochip. The microenvironment of the modification process, such as the ionic strength (pH) of the buffer solution, is one important factor affecting the immobilization of ZEN–OVA on the chip. A certain concentration of ZEN–OVA solution (29.4 μg mL^−1^) with acetate buffer (0.01 mol L^−1^) of different pH (3.5, 4.0, 4.5, 5.0, 5.5) was prepared to evaluate the pH effect on ZEN–OVA immobilization onto the chip surface.

**Figure 2:**
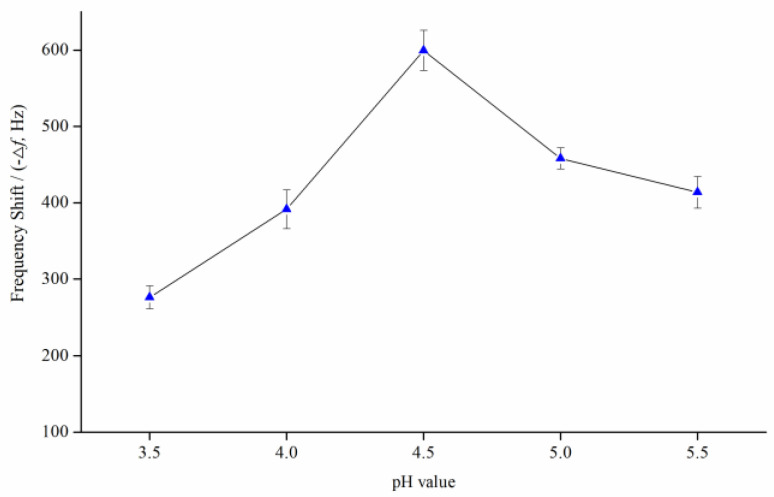
The resulting frequency shift during the immobilization of the ZEN–OVA conjugate at different pH values.

As illustrated in Figure 2, the shift of the oscillation frequency of the QCM chip showed significant differences under different pH values of buffering solution. There was a gradual increase (275.3–599.2 Hz) in the frequency signal, with an increasing pH from 3.5–4.5. This implied an increase in immobilization of the ZEN–OVA conjugate to get a maximum at pH 4.5 (599.2 Hz), which represents the maximum detected mass of ZEN–OVA. Then, a gradual decrease (599.2–413.5 Hz) appeared with a rise of pH from 4.5–5.5, which implied that increasing the pH led to a lower rate of immobilization. As a result, pH 4.5 was considered to be the optimum condition for achieving maximum immobilization on the chip surface. A larger frequency change meant more ZEN–OVA immobilized on the chip surface (approximately 643 ng on one chip at pH 4.5), leading to a wider signal response range and a lower detection limit. Therefore, the acetate buffer at pH 4.5 was applied for the modification of ZEN–OVA conjugates on the QCM chip surface.

### 3.2. Optimization of the Anti-ZEN Ab Concentration

**Figure 3:**
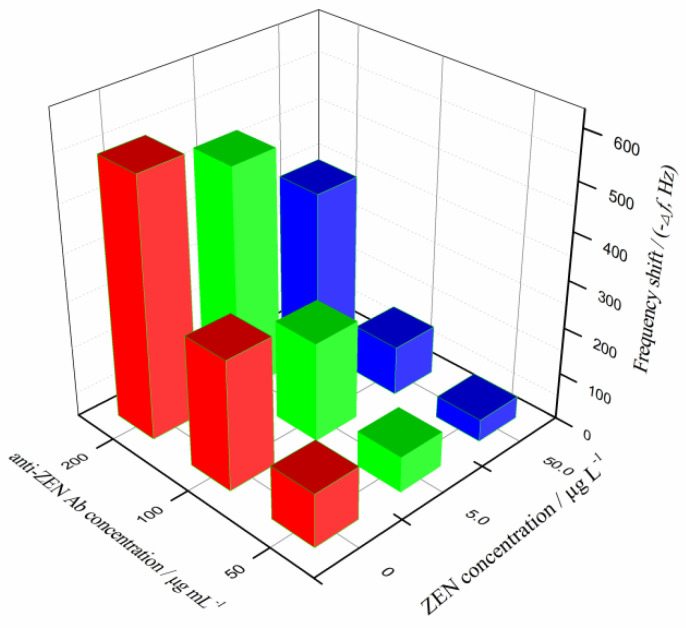
Comparison of frequency shift at the tested ZEN concentrations, using different concentrations of anti-ZEN Ab.

The concentration of Ab is another crucial experimental parameter in immunoassay, which determines the sensitivity, detection limit, and detection cost of immunoassay. The study compared the results under three tested Ab concentrations (50, 100, and 200 µg mL^−1^) for different ZEN concentrations (0, 5.0, and 50.0 µg L^−1^). The results are shown in Figure 3.

In the absence of ZEN, the increasing concentration of anti-ZEN Ab causes a gradually increasing frequency change, ranging from 113.4 to 548.4 Hz, which indicates that the Ab effectively binds to the coating of ZEN–OVA conjugates on the chip surface. With the gradual increase of the added ZEN concentration, due to the competition with the coated antigen for the Ab, the binding amount of anti-ZEN Ab on the chip surface decreases (the frequency change appears to be significantly reduced). In the ZEN concentration range tested in the experiment, the chip frequency changes caused by three different Ab concentrations were 47.2–113.4 Hz (50 µg mL^−1^), 104.5–275.6 Hz (100 µg mL^−1^), and 352.6–548.4 Hz (200 µg mL^−1^), respectively. This was obviously related to the amount of anti-ZEN Ab protein that could freely and specifically bind onto the chip surface. However, the excessive amount of Ab required would greatly increase the detection cost, which was not conducive to the application of the constructed QCM immunochip. By comparison, the anti-ZEN Ab concentration of 100 µg mL^−1^ was finally selected for ZEN piezoelectric immunoassay.

### 3.3. Regeneration of QCM Immunochip

**Figure 4:**
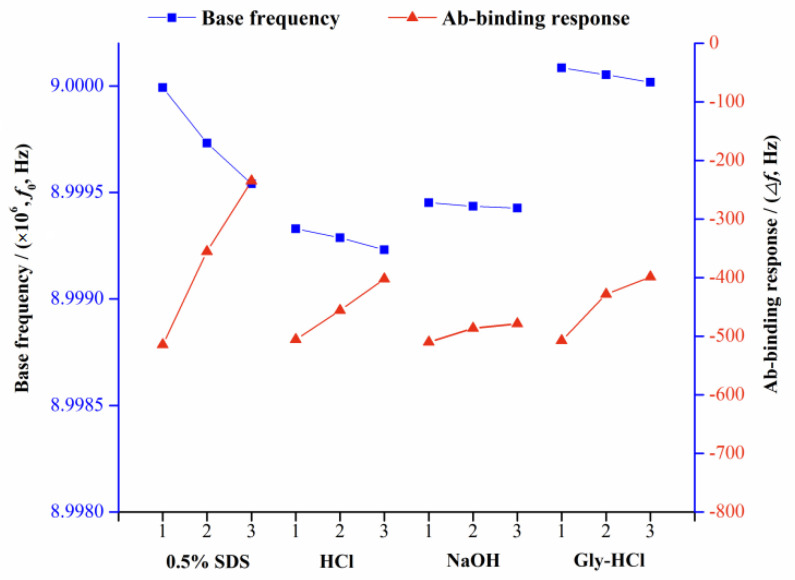
Results for the base frequency and Ab-binding response obtained using various regeneration solutions. The number on the *X*-axis represents the analysis cycle.

To evaluate the regeneration of the developed QCM immunochip, different properties of solutions including 0.1 mol L^−1^ HCl, 0.01 mol L^−1^ Gly-HCl (pH 1.5), 0.05 mol L^−1^ NaOH, and 0.5% SDS were tested to dissociate the bound anti-ZEN Ab from the chip surface. Figure 4 illustrated the resulting changes of the base frequency and Ab binding response, using these regeneration agents.

As illustrated in Figure 4, it was observed that when HCl, SDS, and Gly-HCl were applied for chip regeneration, the base frequency of the QCM chip was significantly reduced, and the value difference between three consecutive tests reached 974.0, 4514.3, and 674.6 Hz. This was because the above three regeneration reagents damaged the pre-modified ZEN–OVA layer, causing part of the ZEN–OVA conjugates to be eluted from the chip surface. In three consecutive binding experiments, the chip frequency change caused by the same amount of Ab (concentration) also decreased. When 0.05 mol L^−1^ NaOH was used as a regeneration reagent, the base frequency change obtained by three consecutive cycles of regeneration was only 247.3 Hz; the average binding frequency of anti-ZEN Ab was 491.6 Hz, and the standard deviation was less than 5%. Based on these above experimental results, 0.05 mol L^−1^ NaOH solution was finally selected for the QCM chip regeneration.

### 3.4. Measuring ZEN Using the Developed QCM Immunochip

**Figure 5:**
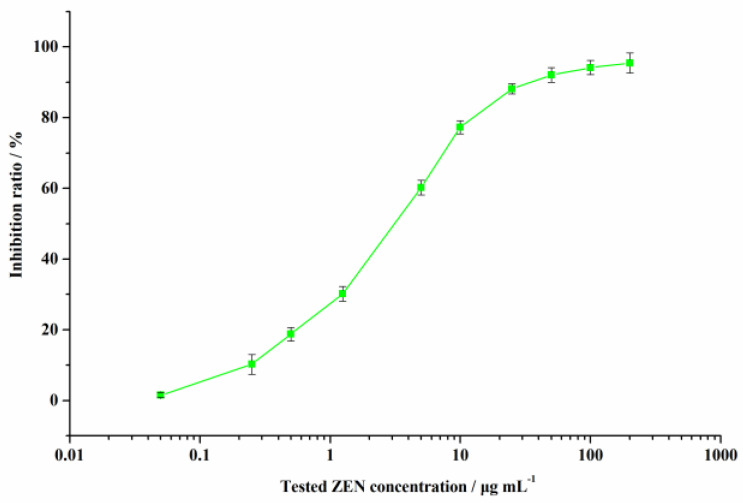
Standard curve for ZEN contaminant detection using the developed QCM immunochip.

The QCM immunochip works according to the principle of competitive reaction between antigen and Ab. A series of ZEN testing solutions (0.05–200.0 µg L^−1^) was evenly mixed with anti-ZEN Ab and uploaded onto the QCM chip surface. A standard curve of inhibition ratio versus tested ZEN concentration on the chip frequency change is illustrated in Figure 5.

Under the optimal conditions, the developed QCM immunochip showed a good response for ZEN with a lower detection limit (IC_15_) (0.37 µg L^−1^) and a higher sensitivity (IC_50_) (3.41 µg L^−1^). The cross-reactivity percent (CR%) for ZEN was calculated using the IC_50_ value for other mycotoxins, under the optimal conditions. The CR% value for ZEN structural analogues was 33.07% (α-zearalenol), 20.62% (β-zearalenol), 39.56% (α-zearalanol), 48.85% (β-zearalanol), 1.55% (zearalanone), and for other toxins (AFB_1_, OTA, T-2 toxin, FB_1_, and DON), it was less than 0.01%, signifying the good specificity of the developed QCM immunochip for ZEN. 

The standard deviation (SD) of the resulting frequency shift of analyzing ZEN content at the same concentration, using nine QCM immunochips prepared in parallel was less than 5%. One immunochip was continuously measured 6 times at the same concentration of ZEN content (after NaOH regeneration), with less attenuation of frequency response (<10%). When stored in PBS solution at 4 °C for 15 days, the QCM immunochip could still maintain the frequency response for more than 90% of the time. A measurement process including the steps of sample pretreatment (about 20 min), QCM measurement (5 min), and regeneration (5 min), could be completed within 30 min. These results demonstrated that the QCM immunochip developed in this research had the merits of good stability and fast speed during preparation, storage, and the application processes, showing good application potential in rapid screening and low-cost detection of ZEN contaminant.

### 3.5. Analysis of Practical Food Samples

In order to validate the feasibility of the developed QCM immunochip in analyzing ZEN in actual food products, typical food matrices that are easily contaminated by ZEN, including corn powder, wheat flour, soy sauce and milk, were chosen and spiked, at three levels—5.0, 50.0, and 100.0 μg kg^−1^ (μg L^−1^). Each measurement was repeated for at least three replicates. Table 1 shows the obtained recovery data in the analysis of spiked ZEN in selected samples, using the developed QCM immunochip and HPLC–MS/MS method.

For all tested samples at the spiked levels, the proposed QCM immunochip obtained an acceptable recovery range of 76.6–92.5%, with an SD of 2.7–6.3% (n = 3) (Table 1), which was relative to the recovery results from the HPLC–MS/MS, with a correlation coefficient of 0.9844. These results indicate that the proposed QCM immunochip has a suitable stability and sensitivity and can detect ZEN in actual food products. Table 2 illustrated a comparison of different strategies for ZEN analysis, which verified that the developed portable QCM immunochip had potential advantages in analyzing ZEN in foods.

## 4. Conclusions

In summary, this work constructed a portable, label-free, QCM immunochip that could effectively detect ZEN contaminant in food products, by combining the convenience of QCM with the high specificity and sensitivity of immunoassays. This proposed QCM immunochip has an acceptable accuracy (76.6–92.5%) and a higher sensitivity (LOD: 0.37 μg L^−1^) in selected food samples, can be reused at least 6 times, and at least one analysis can be finished in 5 min, exhibiting the merits of rapid, accurate, sensitive, and low-cost detection. The construction and working method of the QCM immunochip proposed in this work can also be used for other target analysis in other fields.

## Figures and Tables

**Table 1:** Recovery results of ZEN from spiked samples determined by the proposed QCM immunochip and HPLC–MS/MS.

Sample	Spiked Levels (μg kg^−1^ or μg L^−1^)	QCM Immunochip		HPLC–MS/MS	
		Recovery (%)	SD (n = 3)	Recovery (%)	SD (n = 3)
Corn powder	5.0	86.3	4.2	87.8	4.8
	50.0	89.0	3.9	90.5	2.6
	100.0	90.6	3.3	92.3	3.9
Wheat flour	5.0	85.8	4.5	87.8	6.6
	50.0	91.4	3.9	92.2	4.7
	100.0	92.5	2.7	93.5	5.2
Soy sauce	5.0	76.6	4.9	79.0	4.0
	50.0	81.0	4.7	83.3	4.3
	100.0	86.8	6.3	87.2	6.3
Milk	5.0	78.8	5.6	80.1	4.2
	50.0	86.4	3.4	86.7	3.9
	100.0	89.0	6.2	90.5	3.8

**Table 2:** Results of the comparison of different reported methods for ZEN detection.

Methods	LOD (μg L^−1^ or μg kg^−1^)	Required Time	Reuse Times	Samples	Reference
Ultra HPLC-MS/MS	0.05	7 min	-	Chinese yam, coix seed	[15]
GC-MS	0.01	1 h	-	Vegetable oil	[17]
Fluorescent -ICAs	0.1	20 min	1	Maize, wheat, vegetable oil	[18]
AuSP-ICA and AuNRs-ICA	5.0 and 3.0	10 min	1	Corn; wheat	[23]
Electrochemical immunosensor	1.5 × 10^−4^/0.25	>1 h		Maize/Beer, wine	[30,31]
ELISA	0.15/0.03–0.05	15–30 min	1	Chicken, pork, beef/Wine	[22,36]
Colloidal Au-based ICA	10	15 min	1	Maize, wheat, rice	[37]
Aptamer-based lateral flow test strip	20	5 min	1	Corn	[38]
QCM immunochip	0.37	5 min	≥6	Corn; wheat flour; soy sauce; milk	This work

## Data Availability

The data presented in this study are available on request from the corresponding author. The data are not publicly available due to that further studies on the research are in progress.
